# Supplementary material for: Low circulating adropin levels in late-middle aged African Americans with poor cognitive performance
Source: NPJ Aging. 2023 Nov 9;9(1):24. doi: 10.1038/s41514-023-00122-4 (PMC10636045; doi:10.1038/s41514-023-00122-4)
Supplement: Supplementary file 2 — Supplementary information [file 41514_2023_122_MOESM2_ESM.pdf]

Supplementary Table 1. Age, sex, body mass index, and serum adropin concentrations.

**Characteristics**	**Sample size (n=352)**	Mean ± Std Deviation, range
Age (years)	352	56.6 ± 4.4, 45 to 65
Females (%)	352	66.7%
Body mass index (kg/m^2)	351	31.3 ± 6.9, 14.5 to 55.7
Serum adropin (ng/ml)	352	3.24 ± 1.47, 0.15 to 8.5

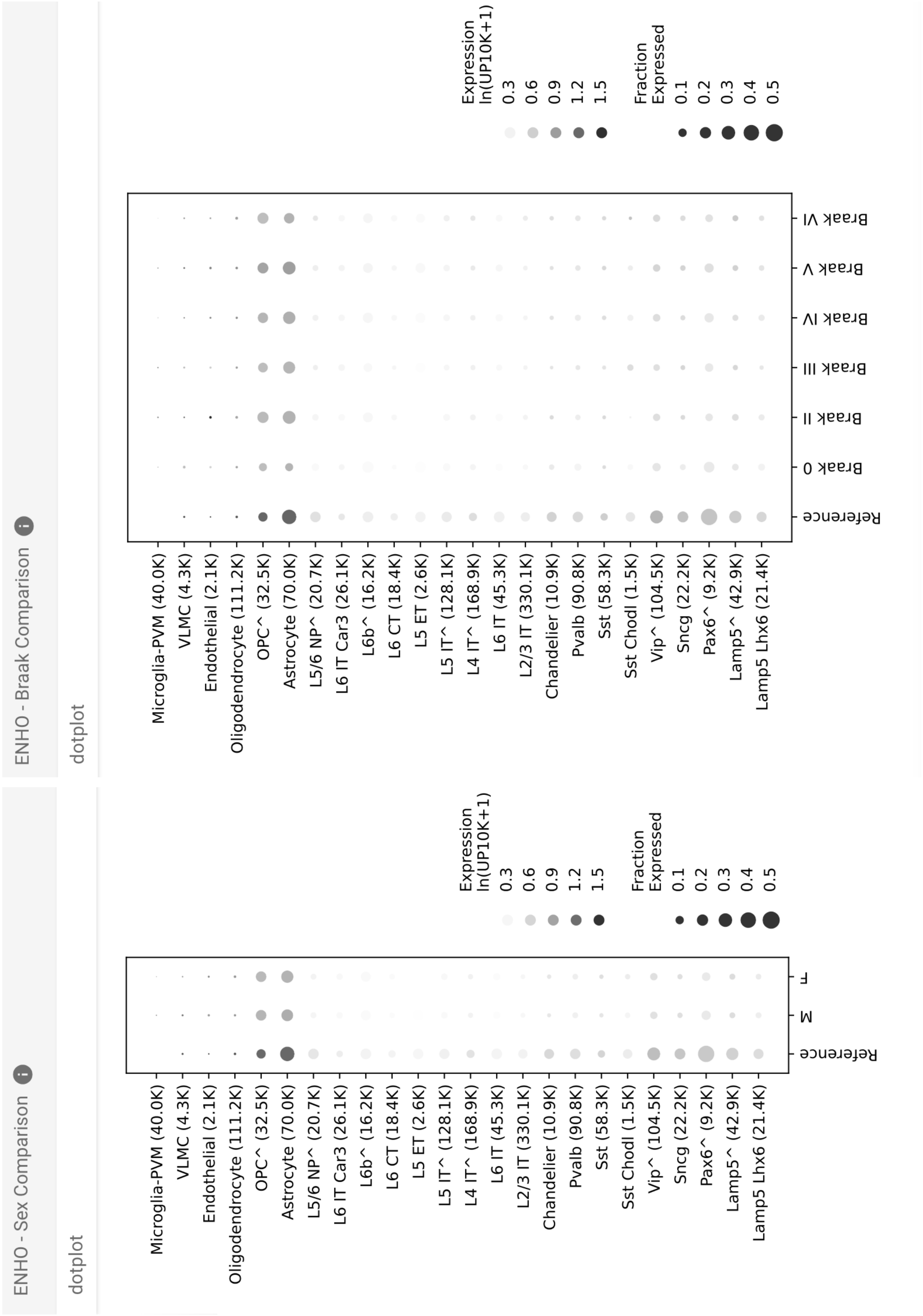

Supplementary figure 1. Comparison of the relative expression of ENHO between cell types in the human middle temporal gyrus (MTG) grouped by sex (A) or BRAAK stage (B).

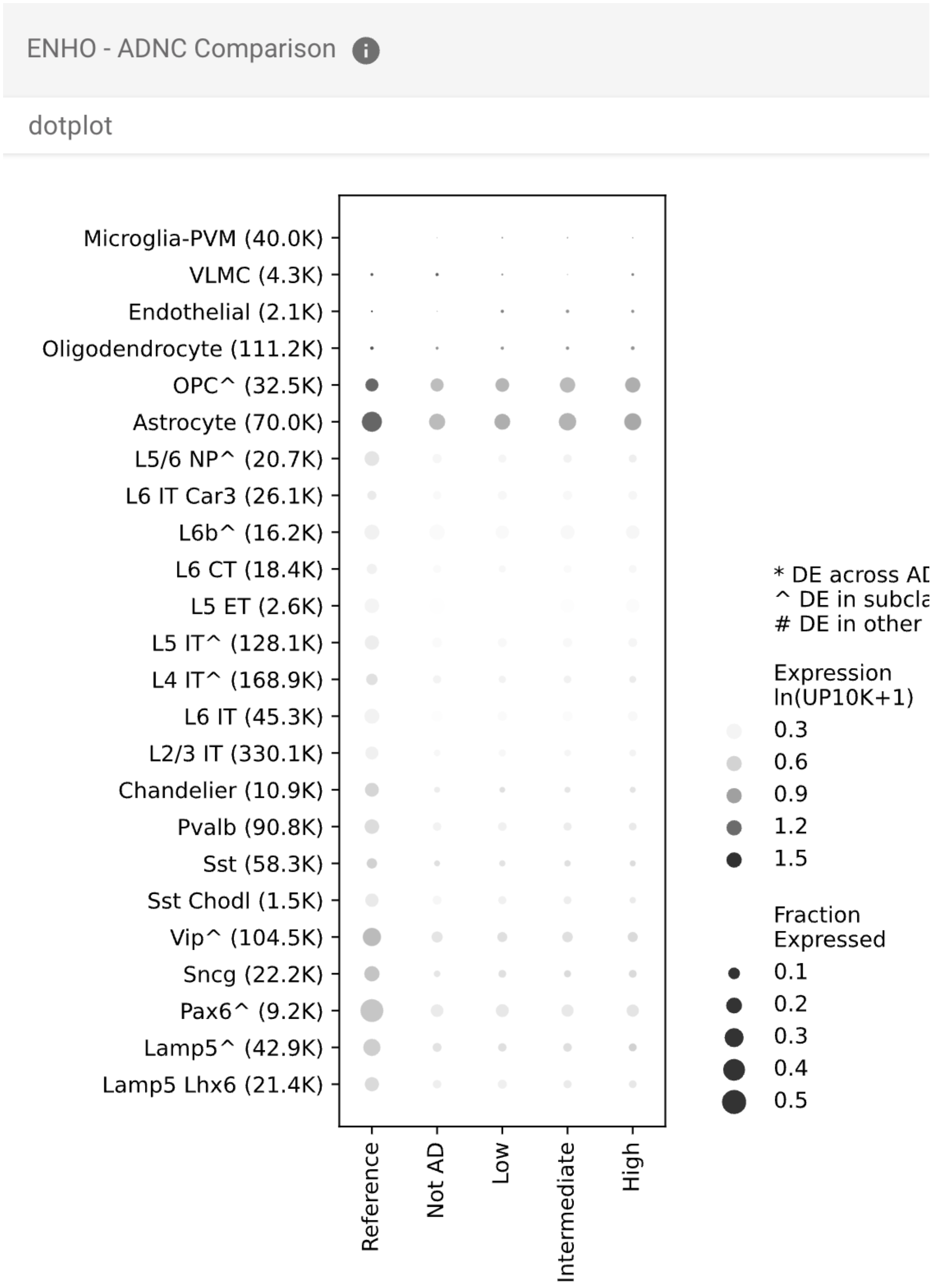

Supplementary figure 2. Comparison of the relative expression of ENHO between cell types in the human middle temporal gyrus (MTG) grouped by ADNC (Alzheimer's disease neuropathologic change) score.

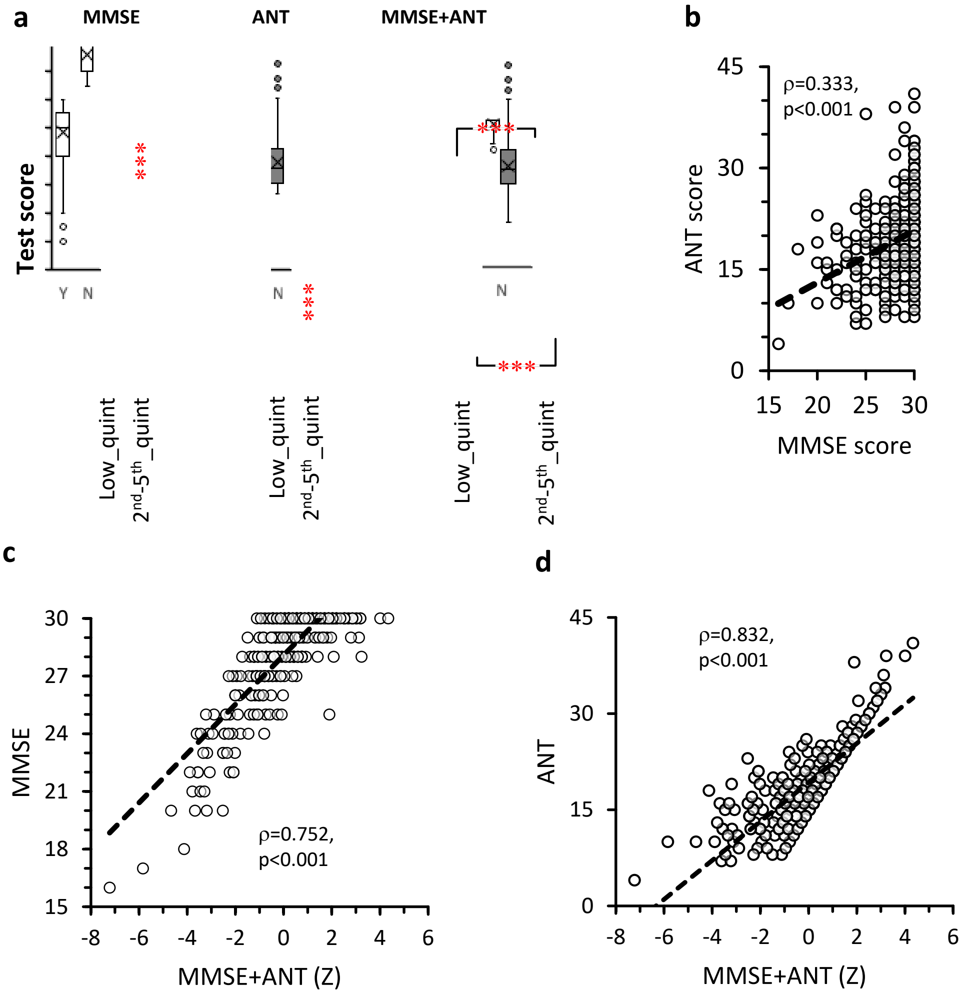

Supplementary figure 3. Distribution of cognitive test results between groups in the low_quintiles for MMSE, ANT, or the composite (MMSE+ANT) scores. (A) MMSE and ANT test score data showing mean and distribution in the worst_quintile and 2nd-5th quintile groups. The left and middle panels show data for the individual tests. The panel to the right shows the results from the individual MMSE and ANT test score data in the composite score low_quint and 2nd-5th quintile groups. (B) Correlation of MMSE and ANT scores. (C) Correlation of MMSE with the composite (MMSE+ANT) score. (D) Correlation of ANT with the composite (MMSE+ANT) score. ***, p<0.001 by Mann-Whitney U test between the indicated groups.
