# Supplementary material for: Low circulating adropin levels in late-middle aged African Americans with poor cognitive performance
Source: NPJ Aging. 2023 Nov 9;9(1):24. doi: 10.1038/s41514-023-00122-4 (PMC10636045; doi:10.1038/s41514-023-00122-4)
Supplement: Supplementary file 3 — Reporting summary [file 41514_2023_122_MOESM3_ESM.pdf]

nature portfolio

Corresponding author(s): Andrew Butler

Last updated by author(s): Aug 7, 2023

Reporting Summary

Nature Portfolio wishes to improve the reproducibility of the work that we publish. This form provides structure for consistency and transparency in reporting. For further information on Nature Portfolio policies, see our Editorial Policies and the Editorial Policy Checklist.

Please do not complete any field with "not applicable" or n/a. Refer to the help text for what text to use if an item is not relevant to your study.
For final submission: please carefully check your responses for accuracy; you will not be able to make changes later.

Statistics

For all statistical analyses, confirm that the following items are present in the figure legend, table legend, main text, or Methods section.

n/a	Confirmed	
☐	☒	The exact sample size (*n*) for each experimental group/condition, given as a discrete number and unit of measurement
☐	☒	A statement on whether measurements were taken from distinct samples or whether the same sample was measured repeatedly
☐	☒	The statistical test(s) used AND whether they are one- or two-sided<br>*Only common tests should be described solely by name; describe more complex techniques in the Methods section.*
☐	☒	A description of all covariates tested
☐	☒	A description of any assumptions or corrections, such as tests of normality and adjustment for multiple comparisons
☐	☒	A full description of the statistical parameters including central tendency (e.g. means) or other basic estimates (e.g. regression coefficient) AND variation (e.g. standard deviation) or associated estimates of uncertainty (e.g. confidence intervals)
☐	☒	For null hypothesis testing, the test statistic (e.g. F, t, r) with confidence intervals, effect sizes, degrees of freedom and P value noted<br>*Give P values as exact values whenever suitable.*
☒	☐	For Bayesian analysis, information on the choice of priors and Markov chain Monte Carlo settings
☒	☐	For hierarchical and complex designs, identification of the appropriate level for tests and full reporting of outcomes
☒	☐	Estimates of effect sizes (e.g. Cohen's *d*, Pearson's *r*), indicating how they were calculated

Our web collection on statistics for biologists contains articles on many of the points above.

Software and code

Policy information about availability of computer code

Data collection	No software code was used.
Data analysis	No software code was used.

For manuscripts utilizing custom algorithms or software that are central to the research but not yet described in published literature, software must be made available to editors and reviewers. We strongly encourage code deposition in a community repository (e.g. GitHub). See the Nature Portfolio guidelines for submitting code & software for further information.

Data

Policy information about availability of data

All manuscripts must include a data availability statement. This statement should provide the following information, where applicable:

- Accession codes, unique identifiers, or web links for publicly available datasets
- A description of any restrictions on data availability
- For clinical datasets or third party data, please ensure that the statement adheres to our policy

Access to datasets from the AAH study are available upon request. The RNA-seq data sets from cultured astrocytes are available from the NCBI GEO database (GSE73721).

Research involving human participants, their data, or biological material

Policy information about studies with human participants or human data. See also policy information about sex, gender (identity/presentation), and sexual orientation and race, ethnicity and racism.

Reporting on sex and gender	Sex was determined based on self-reporting; gender was not investigated. Similar results were found in participants of both sexes.
Reporting on race, ethnicity, or other socially relevant groupings	AAH was a population-based, community-dwelling cohort of self-identified African Americans in 2000–2001 in the Saint Louis metropolitan area.
Population characteristics	The study population includes 358 participants, with 67% being female. The mean age was 57 years, and the mean standardized Mini-Mental Status Examination (MMSE) score of 28.
Recruitment	Recruitment used a multi-stage probability sampling methodology aimed at selecting approximately equal numbers of participants from inner-city and near suburban neighborhoods northwest of Saint Louis City.
Ethics oversight	Saint Louis University

Note that full information on the approval of the study protocol must also be provided in the manuscript.

Field-specific reporting

Please select the one below that is the best fit for your research. If you are not sure, read the appropriate sections before making your selection.

☒ Life sciences ☐ Behavioural & social sciences ☐ Ecological, evolutionary & environmental sciences

Life sciences study design

All studies must disclose on these points even when the disclosure is negative.

Sample size	This study includes all participants of the previous AAH study (n=998) for which serum samples were available (n=358). The RNA-seq analyses was based on previous studies, which had established sample sizes.
Data exclusions	Several samples were excluded based on adropin measurements. Adropin assays were performed in triplicate, and the initial criterion for inclusion was based on the coefficient of variation (%CV). For samples with %CV > 20, the 'outlier' was removed. Data from triplicates with no clear outlier (5 samples) were excluded.
Replication	These findings have been replicated with samples from an independent cohort from the MAPT study in France.
Randomization	N/A
Blinding	N/A

Reporting for specific materials, systems and methods

We require information from authors about some types of materials, experimental systems and methods used in many studies. Here, indicate whether each material, system or method listed is relevant to your study. If you are not sure if a list item applies to your research, read the appropriate section before selecting a response.

Materials & experimental systems

n/a	Involved in the study	
☒	☐	Antibodies
☐	☒	Eukaryotic cell lines
☒	☐	Palaeontology and archaeology
☒	☐	Animals and other organisms
☐	☒	Clinical data
☒	☐	Dual use research of concern
☒	☐	Plants

Methods

n/a	Involved in the study	
☒	☐	ChIP-seq
☒	☐	Flow cytometry
☒	☐	MRI-based neuroimaging

Eukaryotic cell lines

Policy information about cell lines and Sex and Gender in Research

Cell line source(s)	Primary human astrocytes were cultured from small pieces of healthy temporal lobe cortices, as reported in Reference 39 (Zhang Y, et al. Neuron, 2016). Data on sex was not reported.
Authentication	Not reported
Mycoplasma contamination	Not reported
Commonly misidentified lines (See ICLAC register)	Not reported

Clinical data

Policy information about clinical studies

All manuscripts should comply with the ICMJE guidelines for publication of clinical research and a completed CONSORT checklist must be included with all submissions.

Clinical trial registration	N/A
Study protocol	Please see the description in the original report of the AAH study (Miller DK, et al. J Am Geriatr Soc, 2004).
Data collection	Please see the description in the original report of the AAH study (Miller DK, et al. J Am Geriatr Soc, 2004).
Outcomes	The outcome measures include functional measures, cognitive ability, physical performance, depressive symptoms, frailty, sarcopenia, and various laboratory-based blood measures.

This checklist template is licensed under a Creative Commons Attribution 4.0 International License, which permits use, sharing, adaptation, distribution and reproduction in any medium or format, as long as you give appropriate credit to the original author(s) and the source, provide a link to the Creative Commons license, and indicate if changes were made. The images or other third party material in this article are included in the article's Creative Commons license, unless indicated otherwise in a credit line to the material. If material is not included in the article's Creative Commons license and your intended use is not permitted by statutory regulation or exceeds the permitted use, you will need to obtain permission directly from the copyright holder. To view a copy of this license, visit http://creativecommons.org/licenses/by/4.0/
